# Food, home and health: the meanings of food amongst Bengali Women in London

**DOI:** 10.1186/1746-4269-10-44

**Published:** 2014-05-19

**Authors:** Hannah Maria Jennings, Janice L Thompson, Joy Merrell, Barry Bogin, Michael Heinrich

**Affiliations:** 1 Centre for Pharmacognosy and Phytotherapy, UCL School of Pharmacy, University of London, 29-39 Brunswick Square, London WC1N 1AX, UK; 2 School of Sport, Exercise and Rehabilitation Sciences, University of Birmingham, Edgbaston, Birmingham B15 2TT, UK; 3 College of Human and health Sciences, Swansea University, Singleton Park, Swansea, Wales SA2 8PP, UK; 4 School of Sport, Exercise and Health Sciences, Loughborough University, Loughborough LE11 3TU, UK

**Keywords:** Food, Home, Garden vegetables, Food perceptions, Health promotion, Bengalis in London, Ethnography

## Abstract

**Background:**

This paper explores the nature of food and plants and their meanings in a British Bengali urban context. It focuses on the nature of plants and food in terms of their role in home making, transnational connections, generational change and concepts of health.

**Methods:**

An ethnographic approach to the research was taken, specific methods included participant observation, focus group discussions and semi-structured interviews. Thirty women of Bengali origin were mostly composed of “mother” and “daughter” pairs. The mothers were over 45 years old and had migrated from Bangladesh as adults and their grown-up daughters grew up in the UK.

**Results:**

Food and plants play an important role in the construction of home “here” (London) while continuing to connect people to home “there” (Sylhet). This role, however, changes and is re-defined across generations. Looking at perceptions of “healthy” and “unhealthy” food, particularly in the context of Bengali food, multiple views of what constitutes “healthy” food exist. However, there appeared to be little two-way dialogue about this concept between the research participants and health professionals. This seems to be based on “cultural” and power differences that need to be addressed for a meaningful dialogue to occur.

**Conclusion:**

In summary, this paper argues that while food is critical to the familial spaces of home (both locally and globally), it is defined by a complex interplay of actors and wider meanings as illustrated by concepts of health and what constitutes Bengali food. Therefore, we call for greater dialogue between health professionals and those they interact with, to allow for an enhanced appreciation of the dynamic nature of food and plants and the diverse perceptions of the role that they play in promoting health.

## Background

### Introduction: Lili

The account below is a brief overview of the first author’s encounter with Lili^a^ (a research participant, FM1), her family, her foods and plants. The issues raised by her story are key themes explored in this paper. Lili’s relationship with the garden and food illustrate how the mediums of plants and foods are important to the home and home-making, as well as serving to create linkages with family and communities both locally and transnationally.

*I first met Lili in East London tending to vegetables on her allotment plot. Surprised by my ability to communicate with her in Bangla and my interest in her gardening, she was eager to show me what she was growing and share the fruits (quite literally) of her labour. Lili soon invited me to her home where I spent several enjoyable afternoons being fed rice and curry and chatting to her two daughters. Lili’s daughters do not share their mother’s same interest in gardening. They do enjoy her food though; the oldest daughter, now married and moved out of home, says her cooking is different to her mum’s “a bit of everything really, a variety of Bengali….English and other dishes…today I’ve brought over some* jolof *rice (a West African rice dish) and Moroccan eggs I cooked”. Lili tells me that the food she cooks with her home-grown vegetables is good for me as the vegetables are “fresh” and “full of vitamins”. Many of the seeds for the vegetables she grows are sent from Bangladesh. Relatives also send her snacks and* shutki *(dried fish) when possible. Although she has only visited Bangladesh twice since moving to the UK in the 1980s, Lili maintains contact with her family through telephone calls and the exchange of gifts. Though London is now her “home” and the place she raised her four children, the continued presence of her “home” in Bangladesh is evident not least through her food and plants.*

Drawing on ethnographic, qualitative research this paper explores how plants and food from *desh* (homeland, Bangladesh) play a crucial role both in home-making in the UK for two generations of British Bengali^b^ women and their role in maintaining on-going links with “home” in Bangladesh (as defined by the research participants, discussed later in the paper). The paper subsequently examines how and why diets change over time and post-migration to the UK, specifically focusing on the changing nature of diets due to the impact of health messages and perceptions of what constitutes “healthy food”. The paper then examines perceptions of food, particularly “Bengali” food, in terms of health. While the findings illustrate consistencies between the research participants’ and professional views of “healthy” food, they also highlight a power imbalance between research participants and community workers/health professionals and the labelling of “Bengali” food as “unhealthy”. We therefore suggest a need for greater two-way dialogue, with multiple understandings of food and health being appreciated, understood and expressed. Finally, the paper explores lay perceptions of health among British Bengali participants before drawing conclusions. As an introduction, we first highlight background literature and an overview of the methodology used.

### Bengalis in the UK

The flow of people from largely rural Sylhet, North-eastern Bangladesh, to urban Britain is connected to the expansion of the British Raj and trade; it was maintained through a process of “chain migration”. Travel between Sylhet and the UK can be traced to the 19th century, with many Sylhetis working as *lascars* (sailors) and therefore travelling to and occasionally settling in the UK [1]. Post World War II, large numbers of Sylhetis started to work and settle in the UK. Throughout the 1960s and 1970s Sylhetis found unskilled employment in factories, textiles and the growing “Indian restaurant” industries; British-based Sylhetis assisted those based in Bangladesh to migrate through credit, arranging documents and places to stay [2,3]. Throughout the 1970s wives and dependents started to join the men [4], the 1980s saw more significant numbers of wives and children moving to the UK, giving a sense of permanency to the community [5,6]. Less is known about migration today; the literature suggests that Bengalis (not only from Sylhet) come for work, to study and marry [7]. According to the 2011 census, the British Bengali population is 447,201, with 50% of the population resident in London [8]. This is an increase of just over 50% from the previous census in 2001. Undoubtedly Bengalis have already made an impact on the British diet through the curry trade, as over 80% of Indian restaurants in the UK Bengali owned [9] and the “curry” is now a favourite and well known part of the British foodscape. However, the curries prepared and served by men in restaurants are very different to the food cooked by women in Bengali homes, which is where this research is concentrated.

Social and ethnographic research provides important insights into the Bengali community regarding identity [5,10,11] and health beliefs systems [12-14]. Gardner has researched and written extensively about Sylhet and the impact of migration to the UK [2,15], and has carried out interesting qualitative research on age, narrative and migration among Bengalis in the UK [6]. Scientific discourse on Bengalis in the UK in regards to food and diet highlights the prevalence of diabetes and other diet-related diseases [16-20]. Given the high rates of type 2 diabetes amongst Bengalis, it is not surprising that there has been a focus on research related to diabetes in terms of health beliefs [21,22] and more specifically food beliefs [23]. Lofink [24] carried out ethnographic research in East London looking at the multiple factors that influence eating habits among Bengali adolescent girls. Within the last decade outside of the UK there has been research conducted exploring issues of food and identity in relation to diaspora in industrialised countries from the disciplines of anthropology, geography and food studies [25-30]. However, there has been no research on food or plants as place-making in the British-Bengali context or related to on-going links to Bangladesh. There is also a lack of research exploring the health values and perceptions of Bengali food. This paper addresses some of these existing gaps in academic literature as well as building on earlier research.

## Methods

This paper is based on ethnographic fieldwork conducted by the first author (referred to as the researcher) as part of her PhD research examining the therapeutic uses of food-plants and the transmission of knowledge among women of Bengali origin in London, Cardiff and Sylhet. For the purpose of this paper, we concentrate on the findings from fieldwork conducted in London specifically related to food. Research in London took place in two stints: January-December 2010 and June-December 2011. A range of methods were employed including focus group discussions, detailed open ended discussions, semi-structured interviews and participant observation. The research in London was conducted primarily among 30 women. The research focuses exclusively on women due to practical reasons and the nature of the project^c^. However, we did find over the course of the research that women were primarily responsible for the cooking and preparation of food in the home. All participants were of Sylheti origin. Research participants were recruited through community centres and a city farm (a space in an urban area working with people, animals and plants run by the community). Snowballing techniques were used, as snowball sampling is an effective way of selecting cases within a network [31]. As the research was in-depth and qualitative, it was concerned with researching specific networks as opposed to a large representative sample. Twenty of the participants were “older”, over the age of 45 and had migrated to the UK as adults. Where possible the researcher made contact with their daughters, all but one of whom had been born in the UK or migrated under the age of five (referred to as “younger participants”).

In order to gain access to Bengali women, the researcher initially volunteered at two community centres (referred to as the Shapla and Asha centres). Early on in the research period (February-March 2010) three focus group discussions were conducted at the Asha centre. The purpose of a focus group discussion is to explore attitudes, perceptions, feelings and ideas about a topic [32]. Ideally the discussion involves between six and nine people and lasts up to two hours, there is a focus to the discussion, the interaction is noted and the researcher will facilitate the discussion [32]. The method has the underlying premise that the attitudes and beliefs of individuals are not formed in a vacuum but are influenced by those around them, [33] allowing groups to explore processes which are jointly constructed [34]. They are often more relaxed than one-to-one interviews [33], and encourage participation from those who may be reluctant to be interviewed alone [34]. The focus group discussions conducted at the Asha centre lasted an hour, were facilitated by the researcher and each discussion had a different theme. Four to six women attended each discussion, with a total of twelve participating. In addition to conducting focus group discussions the researcher regularly attended the centres as a participant observer and recruited interviewees.

At the community centres and the city farm the researcher acted as a participant observer. Participant observation is the process of immersing oneself in a “culture” through observing and participating in everyday activities of people in their natural setting and intellectualising what has been seen and heard [35]. Establishing rapport and engaging in conversations are important aspects of participant observation. Both the Asha and Shapla community centres ran weekly two-hour health and social sessions primarily for older Bengali women, up to thirty women attended the Shapla centre sessions and fifteen the Asha centre sessions. During the research period (January-December 2010 and June-December 2011) the researcher attended both sessions weekly, interacting with the women at the groups. She also visited a city farm in East London at least one afternoon a week during “growing season” (March-September); the farm had several allotments that were used by Bengali women and men. Time on the farm was spent observing and interacting with allotment holders. Ten Bengali women shared their experiences and discussed plants and their uses.

From the community centres and networks in Bangladesh, eight women (three mother and daughter pairs and two additional interviewees) were formally interviewed at home using a semi-structured interview guide, developed following initial research at the community centres. Semi-structured interviews take less time and are more focused than unstructured interviews [36]. They follow an interview guide which identifies a list of issues with probes, however it is flexible and allows one to talk in depth about topics of interest [32]. The interview guides covered five broad topics (health beliefs and practices; food; therapeutic plants; transnational ties and generational change). The interviews lasted between thirty minutes and an hour each. The interviews were piloted in September 2010. They were adjusted accordingly and were conducted during the researcher’s second stint of fieldwork (June-December 2011).

Ethical approval for the research was granted by the London School of Pharmacy and informed verbal consent was given by the research participants. Focus groups and interviews were audio recorded and transcribed verbatim and during more informal interactions, detailed field-notes were taken. The findings were analysed using a thematic approach and with the assistance of the computer software HyperRESEARCH (Randolph, USA). The researcher “immersed” herself in the data before identifying themes which were categorised and coded, the data were analysed through the development of concepts and linking themes and finding patterns [33]. HyperRESEARCH enabled all the fieldnotes and transcriptions to be uploaded onto one platform, in which pre-defined themes could be highlighted and collated, thus making them easier to analyse.

Research that is valid means that the instruments of research, the data generated and the subsequent findings are both accurate and trustworthy [36]. In order to ensure the data were valid a number of measures were taken. They included the researcher adopting a reflexive approach to the research and reflecting on her role as a researcher throughout the research process [32,36]. When conducting the research she strove to build relationships in order to make the participants feel comfortable and gain accurate information [37]. The transcriptions were checked for accuracy by a native Sylheti speaker. Detailed field notes were maintained and multiple research methods employed enabling the cross-verification of data [32]. Data were checked for incomplete and inaccurate information by the researcher continuously reviewing field notes throughout the research and writing up period [36]. Any missing data would be followed up on and inconsistencies would be checked by contacting the participants and/or checking on other data and background information. Finally, when recording the information, direct quotes and raw data were used as much as possible [38].

## Results and discussion

The findings presented in this paper focus on several key themes. The first theme examined is the role of food, particularly Bengali food, in connecting the participants to “home” in Bangladesh. Changes in food consumption are also examined. The second theme is the role of garden plants regarding on-going connections to Bangladesh. Subsequently, the third theme the paper examines is perceptions of Bengali food in terms of health. The final theme explored is lay perceptions of healthy food according to the participants of the study.

### ‘Bengali’ food: connections to ‘home’ and changes

The food explored in this paper is food found in the “home”. “Home” refers to both the physical structure of a home and a symbolic site of belonging, embedded in historical and geographical relations and meanings [26,29]. The participants in this research refer to both “home” in the UK and “home” in Bangladesh. The two homes are not exclusive of each other as they are linked both in memory and translocal on-going connectedness. Home-making is described by Bhatti and Church [39] as “an active process sought to capture individual agency and household practices in constructing and constituting the meaning and value of the home”. Closely related place-making in the context of the paper refers to “the process that transforms a space into a place, from the abstracted unfamiliarity associated with the idea of space into a place, familiar and meaningful” [40]. Place-making can incorporate home-making, however it is concerned with space in general and not specifically the household. Like home, home- and place-making are dynamic, engaging with the inside and outside worlds. Foods bought, transferred, grown and cooked serve to illuminate and define meanings of home and the people who inhabit home as well as those they interact with. Indeed food is an embodied and visceral experience that can powerfully create and re-create “home” and “memory” through multi-senses [30] and has been highlighted as a carrier of nostalgia, tradition and identity [41]. Sutton [42] argues that food is a “cultural site” through which worlds displaced in space and time can be imagined through tangible means. Food is highly symbolic, embedded in history and social structures yet malleable as both food and the people who consume food change. Our research highlights how food is not only place-making and indeed nostalgic at times, but is also a fluid and transformable “cultural site”, actively connecting people to transnational communities and being redefined across space, time and generations.

Throughout the course of the research it was evident that food was an important aspect of people’s homes and lives. For example, food was an essential part of all visits, and the researcher would regularly be asked numerous questions about food: What food do you like? Do you like Bengali food? What type of Bengali food? Do you eat fish with bones? Can you eat spicy food? When visiting research participants at home, food was always served; preferably a rice meal, failing that tea and snacks were a minimum (serving no food was not an option). The rice meal that was offered, usually by older participants, would consist of white rice, at least one *baji* (vegetables fried in different spices), a meat and/or fish curry and *daal* (lentils). Salads would occasionally be served. *“These are routine; curry, fish and vegetables”* explained one participant (CD1). Home and “Bengali” food were spoken about interchangeably. There were similar views as to what Bengali food consisted of; “rice and fish” were considered quintessentially Bengali. Spices, lentils, certain vegetables, vegetables fried as *baji* with spices and various *mishtis* (sweets) were described as “Bengali”. Despite the apparent similarities in views as to what consists of Bengali food, there was plenty of room for negotiation. For the younger participants Bengali food was primarily connected to home and mothers. However, for the older participants Bengali food also connected them to “back home” in Bangladesh, not least through the active on-going exchange of food products between the UK and Bangladesh.

Spices, vegetables and fish are found in shops and supermarkets throughout London. In East London particularly there are several shops catering specifically to Bengali tastes, selling food items such as *ilish* (Hilsa fish)*, shutki* (dried fish) and *deshi* (from Bangladesh) chicken. Despite accessibility to an industry catering to Bengali food, all of the participants reported themselves and/or members of their families bringing over foodstuff from Bangladesh. Vegetables, fruit, *paan* (*Piper betle* L.) and its accompanying *shupori/goa* (*Areca catechu* L., betel nut), sweets, snacks such as *pittas* (Bengali flatcakes)*, mouri* (puffed rice), *chanachur* (a spicy snack, sometimes known as bombay mix) and certain spices were all reported to be brought over from Bangladesh. The items sent back are not depended upon as a regular food supply, but rather are sent as and when it is possible. The reasons for these regular exchanges vary across individuals and families.

Some of the items sent from Bangladesh are more difficult and expensive to acquire in the UK, such as seasonal fruit. For example, one older participant (IM2) said she has coconuts and bananas sent from Bangladesh when the opportunity arises. While these are available in the UK, she explained that they are more expensive and not as fresh as those in Bangladesh. Mangos, certain chillies and vegetables not as readily available in the UK are also brought back from Bangladesh. There is also more variety in Bangladesh; for instance, there are various types of *shutki* (dried fish)*,* prepared and fermented differently in Bangladesh. As illustrated, food is important to the home and food brought from Bangladesh enriches the food at home. Food from *desh* (homeland) as the source of Bengali food was considered by participants to be more “fresh” and tastier than food in the UK. While the exchange of these foodstuffs is valued in terms of its taste and “freshness”, another dimension the exchange is that connectedness between the two homes is maintained and strengthened. Gifts such as homemade *pittas* and the snacks *chanchur* and *mishtis* indicate food is sent with emotion rather than only for practical reasons as explained by a participant *“they send us things that we like, homemade things, that’s the differences”* (IM4). “*They send us these things because they love us”* another participant (IM3) succinctly explained. Sutton [35], in his book focusing on food in Kalmyous, examines the importance of food as gifts. He argues that the generosity these food gifts entail are a key site for elaborating group identity and communal memories. Mauss [43], in his influential analysis of “gift exchange”, stresses the reciprocal nature of gift exchanges and their importance in the maintenance of kinship relations. Though there is not the space in this paper to explore in depth the theories of gift exchange, our research did find that gift exchange was mutual and strengthens connections with family in Bangladesh. Upon return to the UK after visiting Bangladesh, participants report suitcases are laden with gifts. Food-gifts are subsequently prepared and consumed, appealing to visceral senses of “home” in Bangladesh and powerful reminders of relations there. While food travels between countries, often in the form of gifts, what is consumed changes over time and across generations.

Before moving onto an examination of garden vegetables, it should be noted that food, particularly food consumed outside the home, was subject to change. During the research younger participants in particular described changes in their diets over the course of their lifespan. Most older participants also described changes in their diet, however they tended to eat less “outside” food than their daughters. While food cooked and consumed at home included Bengali food, cereals, pasta and breads (described as “English” or more frequently described as “Western”) were also eaten at home. Foods eaten outside the home include fast foods such as hamburgers, pizzas and fried chicken. Also observed and described was the consumption of Indian, Middle Eastern and Nigerian food. The incorporation of “outside” food as well as continuing to eat food of one’s parents’ country is consistent with other studies looking at the eating patterns of migrants [25,26,28]. When looking at food in so-called globalised cities, this eclectic mix of foods is not unusual and very reflective of a diverse environment. Dyck [26], in research conducted among Indian immigrants to Canada, argues that what is ingested articulates a dual or hyphenated identity (Indian-Canadian). In many ways this is similar to our findings; with effort taken to acquire “old” foods from “back home”, while concurrently “new” foods are introduced to the home and diets subsequently adapted. In London the “new” food eaten, while sometimes described as “English”, is rarely what would be associated with “traditional” English food but instead is reflective of a global food re-worked, now common to the British food-scape.

Food is important to the home, more specifically Bengali food is important to home “here” in the UK. It is also important to maintaining a connectedness to home “there” for older participants. Food and its associated meanings are re-imagined and changed through migrations and generations; this is both reflected in and serves to define the home. Another type of food that is of critical importance to home making and maintaining links to ‘back home’ is food that is grown by the participants themselves.

### Garden vegetables and home

The growing of food plants brings into sharp focus foods as home-making, parts of memory and links to “home past”. Like Lili (FM1), older participants invested time, effort and money into growing plants and food. They would grow plants on windowsills, in gardens, allotments and community gardens. “Bengali” vegetables grown include *lau (Lagenaria siceria,* bottle gourd)*, lal shak (Amaranthus.)* and other *shaks* (leafy vegetables) and *kochu (Colocasia esculenta,* taru*)*, in addition to those found more commonly in the UK such as tomatoes, potatoes, onions, coriander and, occasionally, strawberries. (See Table 1: plants commonly grown in Bengali gardens in London). The growing of vegetables is a practical act, enabling one to grow food to feed one’s family and acquiring fresh Bengali vegetables that are unavailable in the shop or are expensive. However, gardening is also a social and symbolic act that serves to create and maintain links to home. Clearly a range of varieties and cultivars of many of these species are being used, but it is beyond the scope of this project to study these taxonomic groups.

**Table 1:** Commonly grown plants in Bengali gardens in London In general these are varieties which are derived from the species listed and sometimes crossbred with other spp.)

**Scientific name**	**Bengali name**	**Purpose of plant**	**Additional information**
*Allium cepa* L. (Amaryllidaceae)	*Piyaj*	The bulbs are an essential part of Bengali cooking.	Frequently grown in the UK and readily available to buy.
*Allium sativum* L. (Amaryllidaceae)	*Roshun*	The bulbs are an essential part of Bengali cooking. They are also taken medicinally (for general health, the heart, hypertension, stomach upsets and sore throat).	Frequently grown in the UK and readily available to buy.
*Amaranthus acanthobracteatus* Henr. (Amaranthaceae)	*Lal shak*	The leaves are eaten as a vegetable with rice. It is considered very healthy as “it contains many vitamins, particularly vitamin A”.	It is considered a “Bengali” plant. It is grown frequently in the UK and seeds are transported from Bangladesh.
*Amaranthus blitum* subsp. *oleraceus* (L.) Costea (Amaranthaceae)	*Danta shak*	The leaves are eaten as a vegetable. It is considered good for general health as it is often eaten fresh and contains many vitamins.	Fruits are transported from Bangladesh and it is grown frequently in the UK.
*Aloe vera* (L.) Burm.f. (Asparagaceae)	*Gritikumari*	Consumed as a drink for health benefits and medicinally (general health, skin, constipation etc.)	The plant is grown inside the home and can be bought in the UK. It is also common to Bangladesh.
		It is also applied topically medicinally for eczema and burns.	
*Basella alba* L. (Basellaceae)	*Pui shak*	The leaves are eaten as a vegetable. It is considered good for general health as it is often eaten fresh and contains many vitamins.	It is frequently grown in the UK and seeds are often brought from Bangladesh.
*Benincasa hispida* (Thunb.) Cogn. (*Cucurbitaceae)*	*Chal kumra, kodu*	The fruit and leaves are eaten as a vegetable.	It is frequently grown in the UK
*Brassica juncea* (L.) Czern. (Brassicaceae)	*Sherash*	The oil is very popular for cooking. The fresh leaves are used in cooking.	Occasionally grown in the UK.
*Capsicum annuum* L. (Solanaceae)	*Shukna morich, lal morich, kacha morich*	A range of chillies are grown and added to different food.	Seeds are often transported from Bangladesh and the plant is frequently grown in the UK .
*Capsicum chinense* Jacq. (Solanaceae)	*Naga morich*	This is one of the most popular chillies.	Seeds are transported from Bangladesh and it is gown in the UK. It is particularly associated with Sylhet.
*Colocasia esculenta* (L.) Schott (Araceae)	*Kochu*	The leaves, stems and rhizomes are all eaten in food. Medicinally it is taken in food to promote blood circulation and to treat rashes and other skin conditions.	The plant is occasionally grown in gardens.
			It is considered a “Bengali” plant.
*Corchorus olitorius* L. (Malvaceae)	*Nali shak, pak shak*	The leaves are consumed as a vegetable, it is considered good for general health.	Seeds are frequently sent from Bangladesh and it is considered a“Bengali”vegetable.
*Coriandrum sativum* L. (Apiaceae)	*Dhonya*	The leaves, stalks and seeds are eaten in food. It is very common in Bengal cooking and is also consumed to promote general health and digestion.	The plant and fruits are common in the UK.
*Cucurbita pepo* L. (Cucurbitaceae)	Chinese *khodu*	The fruit, leaves and flowers are eaten in food. As a fresh food it is considered healthy.	Literally translated it is “Chinese pumpkin”.
*Cucurbita maxima* Duchesne (Cucurbitaceae)	*Mishti kumra*	The fruit is eaten as food. As a fresh food it is considered healthy.	It is frequently grown in the UK.
*Lablab purpureus* (L.) Sweet. (Leguminosae)	*Sheem, uri*	The fruit and seeds are often added to curries. They are grown frequently in the UK and valued for their good health.	The seeds are often transported from Bangladesh.
*Lagenaria siceraria* (Molina) Standl. (Cucurbitaceae)	*Lou*	The fruit and leaves are eaten as food. As a fresh food it is considered healthy.	It is frequently grown in the UK.
*Raphanus raphanistrum* subs. *sativus* (L.) Domin (Brassicaceae)	*Mullah*	The secondary hypocotyl-root and bulb are eaten in salads and the leaves and stems cooked as a vegetable; as fresh produce it is considered healthy.	It is frequently grown in the UK.
*Solanum lycopersicum* L. (Solanaceae)	Tomato	The fruit is very common in Bengali food.	It is frequently grown and the seeds bought to the UK.
*Spinacia oleracea* L. (Amaranthaceae)	*Palong shak*	The leaves are consumed as a vegetable, it is considered good for general health.	It is frequently grown in the UK.
*Trichosanthes cucumerina* L. (Cucurbitaceae)	*Chichinga*	The fruit and the leaves are eaten as a vegetable. As a fresh vegetable they are valued for their good health.	It is frequently grown in the UK and seeds are transported from Bangladesh
*Vigna unguiculata* (L.) Walp (Leguminosae)	*Barbati*	The fruit and seeds are often added to curries. As a fresh vegetable they are valued for their good health.	It is frequently grown in the UK and seeds are transported from Bangladesh.

Previous research into gardening highlights gardens as social, personal and symbolic spaces [39,44,45]. Research into reasons for gardening among the UK population has found that gardens are rich sources of social interactions as well as private havens, are sources of engagement with nature and reflect a connection to personal history and identity [39,44,46]. Literature on migrant gardens in industrialised countries points to gardens as sites where memory is encapsulated as a lived and visceral experience, and homeland is captured through tastes, sights and smells [45,47,48]. Similar to earlier research, it is clear from our research that gardening is a powerful means of reminding one of “home past”. It was often through a nostalgic lens that participants in the study spoke about the “*sonar mathi”* (golden earth) of Bangladesh, where plants grew freely with ease. The *sangalis* (wooden pergolas that vegetables grow up) that can be found all over Bangladesh have been replicated, space permitting, by Bengali gardeners in the UK. While practical, they are also a visually powerful reminder of Bangladesh. The fresh produce, particularly the Bengali vegetables grown, were reported by participants as being “tasty” and “fresh” and much better than any vegetables found in UK shops. The garden is a site where memory can be visualised and drawn on through practical action, fulfilling the everyday needs of food. Furthermore, like food, seeds from *desh* were frequently reported to be sent to the UK (all the seeds listed in Table 1 were reported to be brought from Bangladesh, with seeds from the *Amaranthacea, Baesellacae, Malvaceae, Leguminosae* and *Cucurbitaceae* families particularly popular). This again serves to connect people now living in the UK to Bangladesh. Interestingly, upon further investigation in Sylhet, these seeds were often not from Sylhet but from other countries–India, Thailand, China and Malaysia. However, the fact that they were brought to the UK via Bangladesh was clearly important and were said to be better, with more availability than ones found in the UK.

When looking at conceptions of “home” and the garden, the growing of vegetables was important to maintaining and creating social connectedness to “home here” as well as “home past” in Bangladesh. At community gardens and allotments it was not uncommon for whole families to visit, often interacting with other gardeners. The produce from these spaces was valued, as people fed their families the harvested foods and gave friends and neighbours their vegetables as gifts. On one of the researcher’s first meetings with Lili (FM1), she showed her around the allotment plots on the farm pointing out who had managed to grow the biggest *lou* (gourd)*.* While each allotment plot was assigned to an individual, relatives would frequently share a plot and work on the land together or separately (with spouses, siblings and in-laws). Adult children would occasionally accompany their older parents, however it was more common for grandchildren to be brought to the plots where they could run around and play. In people’s homes seeds and plants would also be exchanged frequently and shared. Interactions were not always positive, however. For example on the city farm there were often tensions between plot holders, with reports of people stealing vegetables from neighbouring plots. Echoing previous studies on gardens, interactions between people (positive and negative) stress that the garden is a social space and that they facilitate links to “home here” [45].

While gardening is primarily an act of older participants, it was by no means exclusive to them. When examining younger participants’ gardening habits it is possible to see how concepts of gardening are re-imagined through interaction with society and “home here”. Among the three daughters interviewed, only one (ID3) expressed interest in gardening. However, daughters of the older Bengali women at both the farm expressed an interest in gardening. This conversation with a younger interviewee provides some insight into what may influence younger participants to be interested in gardening:

Researcher: *So why…do you enjoy or do you like the taste of home grown food or?*

ID3: *I think it’s to do with um, I think in our culture, particularly in like Islam as well there’s that emphasis on like kind of pure living, like eating healthy food. You know you’ve got to look at where your food comes from, who made it and things like that…I don’t know what they call it but it’s just that sense of you know, you’ve prepared your own food and you’ve grown your own food, so I think in that sense there’s something special about it*

Researcher: *Ok, so would that be a more Islamic thing or a Bengali thing? Or both maybe?*

ID3: *Um, it might be a bit of both. I think it’s just that natural living, trying to live a natural way of life…um part of it is from Islam as well, that influence, and part of it is from mum because she’s always been growing her own vegetables and things… and then there’s the uh, I think it has become quite big now, even in the media, they’re promoting all of this stuff, like ‘make your own produce’……….*

Yeah, yeah, all this stuff is good…and organic as well like growing your own food and trying to use organic soil.

In her narrative, the younger participant combines beliefs about her “culture” and Islam as well as recognising her mother’s influence while linking in with wider narratives of the benefits of home-grown produce and organic food. For the younger participants that did engage with gardening, they spoke about observing their mothers or accompanying them; however, it is also compatible with narratives of “growing your own vegetables” and organic food production promoted in sections of the media and popular among differing groups. While the link to “home” may be less evident among younger participants, it does illustrates how the different aspects of “home” (family, roots, community) as well as the wider society play out through gardens. Although highly valued at home, it is the interaction with wider society where the value of Bengali food and plants are often devalued, which is now discussed.

### Food, health and power imbalances

Public health messages regarding diet penetrate women’s lives via the media, schools, community centres and GP surgeries. Participants and workers at the community centres reiterated these messages. Dietary advice from health professionals was highly valued and rarely questioned by participants. Furthermore there was generally a consistency between the participants’ views and diet-focused public health messages. Participants classified foods in terms of specific nutrients and food groups (such as protein, carbohydrates, dairy, vegetables, fruit, etc.). Participants generally agreed that one’s diet should include plenty of vegetables and some fruit. They reiterated that oil, *ghee* (clarified butter) and salt should be limited. Two of the interviewees stated that fast food should be avoided. Despite the importance of rice, the participants stated that it was important to reduce rice consumption by having smaller portions and/or having only one rice meal a day. Occasionally there was some confusion over messages; for example during a health session at the Shapla centre there was a debate as to whether red meat should be eaten and how much.

Despite a basic level of knowledge regarding standard nutritional advice, not all participants reported taking health advice, or the extent to which it was taken varied. Food and change to eating habits was frequently spoken about in the context of health conditions. For example, a participant Amira (IM3) reported changing her diet after a diagnosis of diabetes and high cholesterol, she consequently reduced her rice, sugar and oil intake. Evidence suggests that people tend to be motivated to change dietary behaviours for health reasons, particularly once diagnosed with disease but less so for prevention or due to social influence or pressure from others [49]. However, other participants while having a basic knowledge regarding healthy eating appeared to pay little attention to the messages practically. A lack of correlation between “rationale” in terms of biomedical nutritional value and food choice is not uncommon, as there are complex social, political, cultural, economic and physical factors that affect food habits and preferences [50]. While all these aspects are important, the “cultural factor” (or “Bengali food”) was frequently identified by community workers and occasionally mothers and daughters as being the reason for unhealthy diets.

The researcher attended health education sessions at both the Shapla and Asha centre. While health and diet information was reported to be useful by attendees of the sessions, information was delivered by community workers or visiting health professionals in a very top-down manner, with little two-way dialogue. During group sessions, a worker would provide basic health messages and participants would be encouraged to ask questions at the end of the sessions. It was assumed that the worker had the “correct” answer and participants did not express opinions that may not fit in with standard medical advice. This often well-meaning but top down approach to providing health information extended to interactions with health professionals and community workers outside of the group sessions. Furthermore, during sessions the “unhealthy” aspects of Bengali food were often highlighted; participants reported these messages were frequently reiterated by doctors and other professionals they had contact with outside the centres. High levels of oil, *ghee,* salt and sweets were viewed as inherent to Bengali food and were identified as items that were particularly consumed by older participants. This concentration on the negative aspects of Bengali food contributes to a broad view that equates Bengali food with the concept of “unhealthy”. The quotes below illustrate how “Bengali” food is often perceived negatively,

“I know in other Bengali households they use a lot of oil, and they do cook a lot of red meat”

(Younger research participant, ID2)

“With my mother, and you will find with a lot of Bengalis, they are unwilling to change their habits. My mother eats a lot of rice, ghee and meat. The vegetables that are eaten are often overcooked.”

(Son of older research participant, IM5)

“There is a lot of spice and oil in Indian and Bengali food. They need more salads, yoghurt and cucumber dishes.”

(Community worker at the Shapla centre)

Research into Bengali food habits in the UK to some extent agrees with these views. Chowdhury et al. [23] found an increase in meat (particularly red meat), oils, sweets and biscuits in the diet after migration to the UK with vegetables, rice and fish continuing to be popular. Grace et al. [22], when examining diets of Bengalis in the context of diabetes, discusses the importance of “special foods” such as *biryani* (white rice cooked in clarified butter with meat and spices) and *mishtis* (sweets) [22]. During fieldwork women did talk about salt, white rice and oil making food “tasty” and similar to Grace et al. [22]’s findings that “special” food was important for entertainment and social occasions. Thus there is a clear rationale for concern regarding the “Bengali” diet and why “cultural” factors are important to this. However, despite “unhealthy” aspects of the “Bengali diet” (high levels of oil, *ghee,* salt etc.) “Bengali” food is not inherently unhealthy according to standard biomedical advice; it contains fish, plenty of vegetables and fruit, pulses, lentils and seasonings such as garlic and ginger, all of which are recommended by health professionals. Rarely was there mention of the “healthy” aspects of the “Bengali” diet. Furthermore the concept of a unified “Bengali diet” is misleading. While many foods are considered “Bengali”, the nature of “Bengali” food is not static but changing and fluid varying across individuals, families, age groups and across seas. The potential structural, practical and socioeconomic factors that affect one’s diet were not mentioned by health professionals, thereby placing the responsibility for diet on individuals and their “culture”. We suggest that people can place moral undertones on food, and urge caution when labelling food as “healthy” and “unhealthy” especially when attaching these labels to a particular group of people.

When unpicking narratives of “good” and “bad” (or “healthy” and “unhealthy”) food values, particularly in the context of ethnic identities, many issues are illuminated. They are indicative of key power imbalances between and within groups as well as a general lack of dialogue between professionals and the research participants. Moral judgements and disempowerment of individuals through the food they consume has been raised by academics. Foucault argued that power was inscribed on bodies through self-regulation of the body via diet and exercise [51]. Building on Foucaults’ work, Conveney [52] examined the emergence of nutrition as a science and a morality in Western civilisation. Discourses of “good” and “bad” food have moral implications creating “good” and “bad” citizens and parents. When values are attached to ideas of ethnicity and culture there is an added dimension. By racialising health and food there is a danger of creating a different “other”; indeed scientific research has a legacy of justifying difference through research and legitimising oppressive power structures [18,53]. Though more subtle, some of the moral undertones described by Conveney [52] were evident in our research. When observing the continual identification of Bengali food as “bad” as well as top down approaches in delivering health messages with little two-way dialogue, key power differences between healthcare professionals and participants were apparent. Expert, legitimised opinion and top-down messages means the balance of power lies with the professionals. The picture is more complex as many participants and Bengali health professionals also subscribe to the view of Bengali food as unhealthy. Such knowledge is thus taken for granted, lay opinions are not discussed as expert opinion is accepted. Bengali professionals subscribing to the view further legitimises opinions, as they are from within the “culture”. Furthermore linguistic and class differences may further strengthen difficulties in dialogue, translation and interpretation. By labelling Bengali food as “bad” and “unhealthy” with little recognition of the potential benefits or the important place of food in one’s “home”, the food and people in turn are devalued.

We are thus caught in a dilemma. “Cultural” issues and links to home are important to understanding the different diets people consume and to providing inclusive public health services that cater to all members of the community. However by focusing negatively on the diets of other groups, there are very real risks of stigmatising and labelling not only the food people consume, but also the people themselves. Bhopal [53] suggests several considerations that need to be made in order to move away from harmful labelling practices in health research and ethnicity. These include the need to recognise the risks of ethnocentricity in research, to examine the importance of wider socio-economic factors in contributing to poor health, the need to recognise differences between individuals as well as the changing and fluid nature of ethnicity and an overall sensitive approach with a clear rationale [53]. These principles can be applied to working with minority groups including Bengalis. In the context of Bengali women it is vital that their wider socio-economic conditions are considered as well as a sensitive approach to discussion of eating habits. Greater value needs to be placed on lay eating habits and beliefs, as by subscribing and reiterating views of the lack of nutritional value to Bengali food, existing power imbalances are maintained. We recommend a move away from a top down deliverance of public health messages and towards a more interactive dialogue. This paper does not have the space to go into detail as to how a more interactive dialogue can be achieved. However, an important place to start in moving towards dialogue is an understanding of the layered conceptualisations of food in the context of health. This paper looks at some of the lay understandings of health and Bengali food. By exploring these understandings it is possible to see how lay perceptions of health are expressed and can be valued and negotiated.

### Lay perceptions of “healthy” food

In addition to notions of nutritional value, our research identified multiple expressions of dietary advice, goodness and health ascribed to food. Looking first at alternative food groups, we draw on Chowdhury et al’s [23] work on Bengali food groups. This in-depth research found a distinctly “Bangladeshi” food classification system emphasising balance, and with some similarities to Ayurveda. This system classifies foods as “strong” and “weak”, “digestible” and “indigestible”, with different types appropriate for the sick, the old, the weak and the strong. There was limited evidence of this classification system at work in the lives of our participants. When asked about these food categories, participants expressed confusion. The category of “digestible” (or “soft”) food was spoken about by several participants, and “strong” foods by a few. “Digestible” foods were given to people of a certain constitution. For instance, if someone is sick, old or young they are often recommended to eat “soft” food as it is described as easily digestible and therefore easy to eat. Examples of “soft” food include rice made as *jau* (rice boiled to create a semi-liquid consistency), *kitchuri* (rice cooked with lentils and mild spices)*,* milk, lentils and spinach. This food is literally soft in texture and is prepared with little or no spices. For very young children, it is “soft” foods that they are weaned on due to its attributed ease of digestion. For the sick, *kitchuri* is particularly given as it is considered nutritious (especially when vegetables are added) and easy to digest. In contrast, food that is classified as hard or “strong” is suitable for most (the well and not too old). “Strong” foods include rice cooked as *pilou* or *biryani* and meat (particularly beef)*.* These foods are physically more “hard” and richer in oils and spices. Strong foods were rarely spoken about; one or two participants discussed them only when asked specifically about these foods, and they were described in contrast to the examples given for “soft” or digestible foods. Thus, the understandings of “Bengali” food groups expressed by participants in the current study did not match the detail of Chowdhary’s work. This could be because these categories and descriptions are theoretical in nature and are not necessarily how lay individuals discuss these groups of foods in their daily lives.

In regards to participants’ understandings of healthy food, the values of “freshness” and “balance” were expressed as particularly important. Participants spoke about “balance” in terms of having different types of food or “a bit of everything” as well as not eating in excess. This is consistent with Chowdhury et al. [23], as they discuss the Ayurvedic concept of balance being important for their participants in terms of avoiding excess (eating and excreting), suggesting these views have parallels with the biological concept of homeostasis. “Freshness” was particularly valued among participants. “Fresh” food is considered food that is cooked and consumed soon after it is picked, plucked or killed. As food loses its freshness over time, it too loses its “goodness”, “healthiness” and “vitamins”. With food that is bought from shops, it is difficult to know how long it has been since it was alive (or growing), and therefore the freshness is judged to be decreased. Food from Bangladesh was considered “fresh” as compared to food found in the UK; this was most likely because the food came from the source and the amount of time taken to arrive in the UK was known (within a few days). However, food grown by one-self was considered more “fresh”. Growing vegetables among participants (particularly older) was done partly to acquire “fresh” food with many “vitamins”. While vitamins were often referred to in general terms by most participants, members of a gardening club in East London told the researcher more about specific vitamins in different vegetables (e.g. vitamins A and D). The combining of concepts of lay understandings of “freshness” with biomedical concepts of “vitamins” is an example of how knowledge can adapt and incorporate multiple meanings. In addition to the concept of “freshness”, there appeared to be certain food-plants that are particularly valued for their medicinal properties and health benefits. These included Neem (*Azadirachta indica* A. Juss) and *korala* (*Momordica charantia* L.*,* bitter gourd)*.* These vegetables were frequently described as “Bengali” and relate to those found in “traditional” medicinal remedies in Bangladesh.

Among younger participants, the above values of health regarding food were less evident. For example, “soft” food was rarely spoken about and knowledge of food-medicines was less than that of older adults. With regards to “fresh” food, it was generally valued while taking on new meanings. Ruby (a participant in her early 20s born and brought up in London, ID3), for example, said she had learnt about the importance of fresh food from her mother. She now equates it with foods that are “organic,” which is something she has learnt in the UK and feels is important to a healthy diet. Knowledge is dynamic and changing, and the body and self incorporate multiple knowledge sources as illustrated by Ruby and the multiple meanings of freshness when applied to garden vegetables. During a focus group discussion, women expressed the differing forms of knowledge they had acquired as they spoke about “*age kota”* (“previous speak/knowledge”) and “*ekone kota”* (“present speak/knowledge”) in relation to the food that they previously ate and they now eat and know to be healthy. Both forms of knowledge are important and changing. Food, and healthy foods in particular, are embodiments of shared knowledge and various meanings influenced by emotions, family and personal histories.

Findings of differing and lay perceptions of food are consistent with research among other migrant groups. Park et al. [54], in their study of Latino women in New York City, explored concepts of health not being expressed purely in terms of nutritional status but instead through concepts such as “freshness”, “purity” and “naturalness”. Dyck and Dossa [27], in their study of Afghan and South Asian migrants to Canada, found that both groups of women combined health knowledge from their countries of origin with health messages in Canada. The women in our study adhered to different forms of knowledge in varying degrees. The participants of this research were less forthcoming in discussing health outside of biomedical terms; during fieldwork the researcher recorded that she was frequently asked what the “right” answer was, and that basic public health messages would be reiterated by the women. Lay understandings of health were rarely in contradiction and frequently complementary to these messages. While the women had a great deal of knowledge about foods and their potential health properties, they were wary of being outside of “correct” knowledge. This reflects a key imbalance of power and a lack of dialogue on equal terms, as discussed in the previous section of the paper. If meaningful dialogue is to occur between groups of differing levels of power (such as between health professionals and patients, or health promotion experts and community-based groups), it is critical to address these power imbalances by encouraging a respectful mutual exchange of information, honouring differing views and experiences and appreciating the importance and meanings of food (for example food from “home”). While expert knowledge is important, so too is lay knowledge. Both should be equally valued, respected and negotiated. While the practicalities of such an approach are not addressed in this paper, we call for dialogue within the spaces of community centres, GP surgeries and community groups. It is only through dialogue that professionals are likely to understand the dynamic and meaningful nature of the food that people consume, and people are more likely to feel respected and continue to negotiate their food habits in conversation with professionals.

## Conclusions

Food and plants are the embodiment of multiple meanings; this paper illustrates how these meanings are fluid and changing over generations, across individuals, homes and groups. In the context of British Bengali women in London, our research found that “Bengali” food and plants are an important aspect of home “here” in the UK across different age groups. Bengali food is also important in connecting one to home “there” in Bangladesh through regular exchange of foodstuffs and seeds. The nature of the foods consumed is transformable, as we can see through differences between “inside” and “outside” food and changes within food at home. The influences on food by those outside of familial networks are illustrated though conceptions of “healthy” food. When looking at concepts of health and Bengali food we identify several crucial points. The first is that the existence of a power imbalance means that Bengali food is often devalued. The second is that there is a general lack of dialogue between health professionals and lay people which may be indicative of wider power structures as well as practical barriers such as a lack of time and interpreting services. Finally through examining lay understandings of healthy diets we find that there are multiple understandings of what healthy food means.

While this paper does not explore the socioeconomic and structural factors influencing one’s diet and contributing towards power differences, it is important to be aware of their existence. In recognising an imbalance in power we urge for a greater dialogue between community workers, health professionals and those they cater to. Dialogue is different to advice, which tends to be a one-way flow of information from professional/worker to patient/participant. In contrast, dialogue, and even more importantly intercultural dialogue, is a mutual exchange of views encouraging expressions of lay understandings of health and recognising the meaningful nature of food. Food is ultimately the embodiment of multiple and fluid meanings-home here, home there, parents, children, local communities, global communities and health-to name a few. This embodiment needs to be taken into account in such dialogues aimed at groups like the Bengali community in Britain.

## Endnotes

^a^All the names and places in the paper are pseudonyms.

^b^The term “Bengali” refers to people that trace origins back to Bangladesh, formally East Pakistan (1947–1971) and previously East Bengal in India. “Bengali” (normally referring also to people from West Bengal in India) is used as opposed to “Bangladeshi” as “Bengali” is the term used by the participants in this study.

^c^The PhD was part of a larger project, MINA (Migration, Nutrition and Aging Across the Lifecourse in Bangladeshi Families: A Transnational Perspective, projectmina.org), focusing on Bengali women in the UK and Bangladesh.

## Competing interests

We, the authors, declare we have no competing interests.

## Authors’ contributions

HMJ was responsible for undertaking the fieldwork, interviews and analysing the data in the research presented in this paper. She wrote the first draft of the manuscript. MH, JT, JM and BB gave support and advice during the entire research process and contributed towards the writing and editing of the paper. All have approved the final version of this manuscript.
